# Activin A as a Novel Chemokine Induces Migration of L929 Fibroblasts by ERK Signaling in Microfluidic Devices

**DOI:** 10.3389/fcell.2021.660316

**Published:** 2021-05-21

**Authors:** Lingling Jiang, Yan Qi, Xianghan Kong, Runnan Wang, Jianfei Qi, Francis Lin, Xueling Cui, Zhonghui Liu

**Affiliations:** ^1^Department of Oral Comprehensive Therapy, School and Hospital of Stomatology, Jilin University, Changchun, China; ^2^Department of Immunology, College of Basic Medical Sciences, Jilin University, Changchun, China; ^3^Department of Genetics, College of Basic Medical Sciences, Jilin University, Changchun, China; ^4^Department of Biochemistry and Molecular Biology, School of Medicine, University of Maryland, Baltimore, MD, United States; ^5^Department of Physics and Astronomy, University of Manitoba, Winnipeg, MB, Canada

**Keywords:** activin A, fibroblasts, migration, ERK, microfluidic device

## Abstract

Activin A, a member of the transforming growth factor-beta (TGF-β) superfamily, contributes to tissue healing and fibrosis. As the innate tissue cells, fibroblasts also play an important role in wound healing and fibrosis. Herein, this study was aimed to investigate how activin A exhibited regulatory effects on adhesion and migration of fibroblasts. We found that activin A induced the migration of fibroblast cell line L929 cells in transwell chamber and microfluidic device. Activin A also promoted L929 cells adhesion, but did not affect L929 cells viability or proliferation. In addition, activin A induced α-SMA expression and TGF-β1 release, which were factors closely related to tissue fibrosis, but had no effect on IL-6 production, a pro-inflammatory cytokine. Furthermore, activin A elevated calcium levels in L929 cells and increased p-ERK protein levels. Activin A-induced migration of L929 cells was attenuated by ERK inhibitor FR180204. To conclude, these data indicated that activin A as a novel chemokine induced the chemotactic migration of L929 cells via ERK signaling and possessed the pro-fibrosis role. These findings provide a new insight into understanding of activin A in tissue fibrosis.

## Introduction

Fibroblasts are widely distributed in various tissues and actively involved in tissue damage repair, remodeling and fibrosis. Fibroblasts stimulated by degeneration, necrosis or tissue injury are demonstrated to participate in inflammatory response and immune regulation by secreting factors such as nitric oxide (NO), interleukin-1β (IL-1β) and IL-6, and facilitate wound healing by infiltrating to the damaged site, gradually replacing white blood cells (Eming et al., 2014). Fibroblasts are also involved in tissue remodeling in the late stage of inflammation through cell proliferation, adhesion, migration and collagen synthesis (Sun et al., 2014; Burr et al., 2020), while abnormal activation of fibroblasts may result in scar formation and tissue fibrosis (Bai et al., 2015; Nagpal et al., 2016).

As a member of the transforming growth factor-β (TGF-β) superfamily, activin A is widely expressed in a variety of tissues and acts as a multifunctional growth and differentiation factor. Activin A regulates cell proliferation, differentiation, apoptosis and migration in an autocrine/paracrine manner, and plays a critical role in embryonic development, neuroprotection, tissue remodeling and immune response (Aleman-Muench and Soldevila, 2012; van den Ameele et al., 2012; Kaiser et al., 2013; Donovan et al., 2017). As suggested in previous studies, activin A controls the cytokine signaling cascades that drive the inflammatory response (Jones et al., 2007), and regulate the activation of M2 macrophages to affect tissue healing and fibrosis in the late stage of inflammation (Morianos et al., 2019). Furthermore, as an important factor in inducing liver, kidney and lung fibrosis, activin A is reported to be tightly associated with the occurrence and development of numerous fibrotic diseases (Hardy et al., 2015; Hruska et al., 2017; Zhang et al., 2018).

The aberrant overexpression of activin A is frequently detected in the case of inflammation or tissue injury (Sun et al., 2014; Qi et al., 2021). Although our previous study has reported the antagonistic effects of activin A and TNF-α on fibroblast activation (Jiang et al., 2020), the role of activin A in adhesion and migration of fibroblasts is not well characterized. In the present study, the murine fibroblast cell line L929 was utilized to investigate the effects of activin A on adhesion and migration of fibroblasts. We found that activin A promoted L929 cell adhesion, migration and TGF-β1 release in a dose-dependent manner, proving further that activin A was a chemokine for fibroblasts in the process of tissue repair and fibrosis. These findings implicated that activin A may serve as a novel therapeutic target for tissue fibrosis.

## Materials and Methods

### Cell Culture and Treatment

Murine fibroblast cell line L929 (American Type Culture Collection, ATCC, Rockville, MD, United States) was maintained in high glucose DMEM supplemented with 10% fetal bovine serum (FBS) at 37^°^C in a humidified incubator containing 5% CO_2_. L929 cells were seed in 12-well plates and treated with 0, 10, or 20 ng/mL activin A for 2 h, with or without the pretreatment with 1 μmol/L ERK inhibitor (FR180204) for 1 h. Experiments were repeated at least three times with independent cell cultures.

### Reagents

Recombinant human/mouse/rat activin A was purchased from R&D systems (Minneapolis, MN, United States). Recombinant murine SDF-1α (CXCL12) were obtained by PeproTech (Rocky Hill, NJ, United States). MTT and Giemsa stain were provided by Sigma-Aldrich (Oakville, ON, Canada). Fluo-4 was bought from Thermo Fisher Scientific (Ottawa, ON, Canada). Enzyme-linked immunosorbent assay (ELISA) kits for TGF-β1 and IL-6 were obtained from eBioscience (San Diego, CA, United States). Selective ERK inhibitor FR180204 was purchased from Absin (Shanghai, China). Rabbit polyclonal antibody against α-SMA was purchased from Abcam (Cambridge, United Kingdom). Rabbit polyclonal antibody against GAPDH was bought from Absin (Shanghai, China). Rabbit polyclonal antibodies against phospho-ERK1/2 (p-ERK) and ERK were obtained from Cell Signaling Technology (Danvers, MA, United States).

### Real-Time Cell Analysis

Real-time cell analysis (RTCA) is a technology based on the principle of microelectronic biosensor chip, which can realize the real-time analysis of cells without markers in the process of experiment. The RTCA instrument (xCELLigence RTCA S16; ACEA Biosciences, California, United States) was used to analyze the proliferation and adhesion properties of L929 cells. Initially, 50 μl of cell-free culture medium was added to the well of an E16 xCELLigence microtiter plate, which was then inserted into the RTCA device. After 1 min, the background impedance was measured for each well. Subsequently, L929 cells (2 × 10^4^ cells in 50 μl culture medium) were added to each well and cultured for 3 h, and then treated with different concentrations of activin A for another 67 h in the proliferation assay. In the adhesion assay, cells and activin A were added to each well together and cultured for 4 h. Cells were monitored in 5 or 15 min intervals. Each experiment was performed in duplicate and repeated three times. The impedance of the cell sensor was described and measured as the cell index (CI), which reflected the cell activity.

### MTT Assay

L929 cells (2 × 10^4^ cells per well) were seeded into a 96-well plate in triplicate and incubated in 1% FBS-DMEM containing activin A (0, 5, 10, or 20 ng/mL) for 24 h. 10 μl of MTT solution (5 mg/mL) was added to each well and incubated for 3 h at 37^°^C. Then the medium was removed, and 100 μl DMSO was added to dissolve the formed formazan crystals. Each experiment was carried out in triplicate. The absorbance at 570 nm was measured using a microplate reader.

### Transwell Chamber Assay

Chemotactic migration of L929 cells was evaluated by transwell chamber assay as described previously (Yuan et al., 2019). Briefly, L929 cells (2 × 10^4^ cells in 200 μl culture media with 1% FBS) were seeded in the upper chambers (8 μm pore size; Corning, NY, United States). The lower compartments were filled with 500 μl culture media containing activin A (0, 5, 10, and 20 ng/mL) or CXCL12 (100 ng/mL). After incubation for 10 h, the cells on the upper side of chamber were removed with cotton-tipped swabs. Cells that had passed through the insert membranes were fixed with 4% paraformaldehyde for 20 min and stained with Giemsa. Experiments were repeated at least three times. The stained cells were imaged on an inverted microscope and cell numbers were counted in five randomly chosen fields from each chamber.

### Microfluidic Cell Migration Assay

A microfluidic triple docking device (D^3^-Chip) fabricated by the standard photolithography and soft-lithography technique was used as described previously (Xie et al., 2017; Yang et al., 2017). Briefly, the channel geometries in each layer were defined by patterning the SU-8 photoresist (MicroChem Corporation, Westborough, MA, United States) through the photomask on a silicon wafer. The SU-8 master was then molded by polydimethylsiloxane (PDMS) (Slugard 184, Dow Corning) by soft lithography to create the negative replica. Inlets and outlets were punched out of the PDMS replica. The PDMS replica was then bonded to a glass slide by air plasma treatment. The microfluidic channels were coated with 0.4% BSA blocking for 40 min at 37^°^C before cell migration experiments. L929 cells were pretreated with vehicle control [dimethylsulfoxide (DMSO)] or 1 μmol/L FR180204 for 12 h. Then, the unpretreated and pretreated cells were loaded to the three parallel test units of the microfluidic device from the cell inlets and allowed to align in the docking structures. Equal volume of chemoattractant solution and medium were added into the three pairs of source wells to configure different gradient conditions. The conditions included 0, 5, 10, and 20 ng/mL activin A gradients in DMEM with 1% FBS. Each experiment was performed in duplicate and repeated three times. The cell images in the device were captured every 5 h for a total of 20 h. To maintain the gradient, the waste in the outlet was discarded and the appropriate equal volume of chemoattractant solution and medium was supplemented. The device was kept in a 37°C incubator containing 5% CO_2_ between the imaging time points.

### Wound Healing Assay

A scratch wound healing assay was performed to assess the action of activin A on the wound healing ability of L929 cells. Briefly, L929 cells (8 × 10^5^ cells/well) were seeded on a 12-well plate and allowed to reach 90% confluency overnight. Then, a scratch-wound (400 μm wide) was generated in the surface of monolayer cells using a 10 μl pipette tip, and the free-floating cells and debris were removed by washing twice with 0.1 mol/L PBS. Cells were cultured in 1% FBS-DMEM containing activin A (0, 5, 10, or 20 ng/mL). For each group, the images on an inverted microscope were taken at 0 h and 24 h after wounding, and the degree of wound healing was measured by the cell migratory distance. Independent experiments were repeated three times.

### Giemsa Staining

L929 cells were cultured with 1% FBS-DMEM containing activin A (0 or 10 ng/mL) in a 48-well plate. After 24 h, cells were fixed with 4% paraformaldehyde, stained with Giemsa solution, and observed under an optical microscope. Experiments were performed in duplicate.

### Western Blotting

Cells were lysed using the protein extraction reagent (Thermo Fisher Scientific, United States) supplemented with the protease and phosphatase inhibitor cocktail (Thermo Scientific, United States) and 0.5 mol/L EDTA solution. Proteins were quantified using the BCA protein assay kit (Thermo Scientific, United States) following the manufacturer’s instruction. Twenty microgram of proteins were separated by electrophoresis with 12% SDS-PAGE gel, and transferred onto a polyvinylidene difluoride membrane. Membranes were blocked in 5% BSA-TBS for 2 h at RT, and then incubated with the primary antibodies overnight at 4^°^C. Membranes were further incubated with secondary antibody conjugated with horseradish peroxidase for 2 h at RT followed by ECL detection (GE Healthcare, United Kingdom). Finally, membranes were scanned with LAS-4000 luminescent image analyzer (Fujifilm, Japan).

### Enzyme-Linked Immunosorbent Assay for TGF-β1 and IL-6

The supernatants of cultured L929 cells were collected, and the levels of TGF-β1 and IL-6 were determined by ELISA kits according to the manufacturer’s protocol.

### Calcium Flux Assay

L929 cells were incubated in 1% FBS-DMEM medium with 4 μmol/L Fluo-4 in dark for 40 min at 37^°^C. Cells were washed twice using the 1% FBS-DMEM, and Fluo-4-loaded cells were recovered in the incubator for another 30 min in dark. Then the cells were divided into different tubes for analysis by flow cytometry. Fluo-4 signal was first recorded for 1 min to obtain the baseline fluorescence signal (F_0_). Cells were stimulated with 0, 5, 10, or 20 ng/mL activin A. The Fluo-4 signal of simulated cells (F) was recorded for another 3 min. The experiment was repeated at least three times. FlowJo software (FlowJo LLC., Ashland, Oregon, United States) was used to analyze the kinetics of Fluo-4 signal intensity. The Fluo-4 intensity was normalized to the baseline for comparison (F/F_0_).

### Statistical Analysis

All data were presented as mean ± standard deviation (SD). Statistical analysis was performed using Student’s *t*-test or one-way ANOVA followed by Tukey’s multiple comparisons test. Difference at *P* < 0.05 was considered to be statistically significant.

## Results

### Effects of Activin A on Adhesion and Proliferation of L929 Cells

**FIGURE 1:**
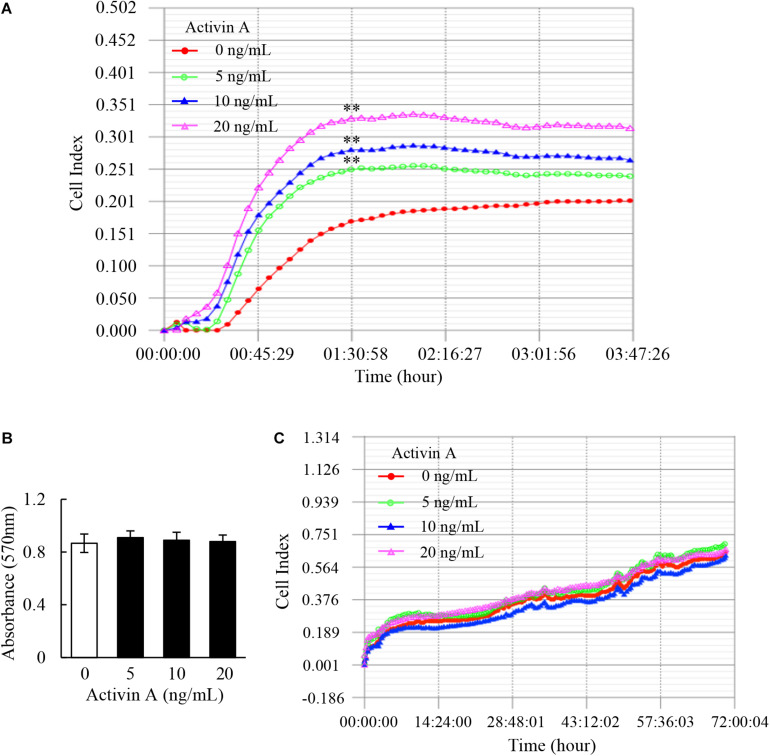
Effects of activin A on adhesion and proliferation of L929 cells. **(A)** Real-time cell adhesion was assessed by RTCA in L929 cells subject to activin A at different concentrations for 4 h. ^∗∗^*P* < 0.01 compared with the 0 ng/ml control group. **(B)** MTT assay was performed to assess the viability of L929 cells treated with activin A at different concentrations for 24 h. Data represent mean ± SD (*n* = 6). **(C)** Real-time cell proliferation of L929 cells was assessed by RTCA in 70 h.

As one of the basic cell activities, adhesion is involved in the cell movement and tissue damage repair. Hence, RTCA was conducted to examine the effect of activin A on the adhesion of murine fibroblast cell line L929 cells. The results showed that activin A significantly promoted the adhesion of L929 cells in a dose-dependent manner (Figure 1A).

The viability and proliferation of fibroblasts can affect cell adhesion and movement. This study further explored the effect of activin A on viability of L929 cells by MTT method. The results showed that activin A had no significant effect on the viability of L929 cells (Figure 1B). In addition, RTCA results showed no significant action of activin A on proliferation of L929 cells (Figure 1C). These results indicated that activin A promoted L929 cells adhesion, but had no impact on the viability and proliferation of L929 cells.

### Effects of Activin A on Migration of L929 Cells

**FIGURE 2:**
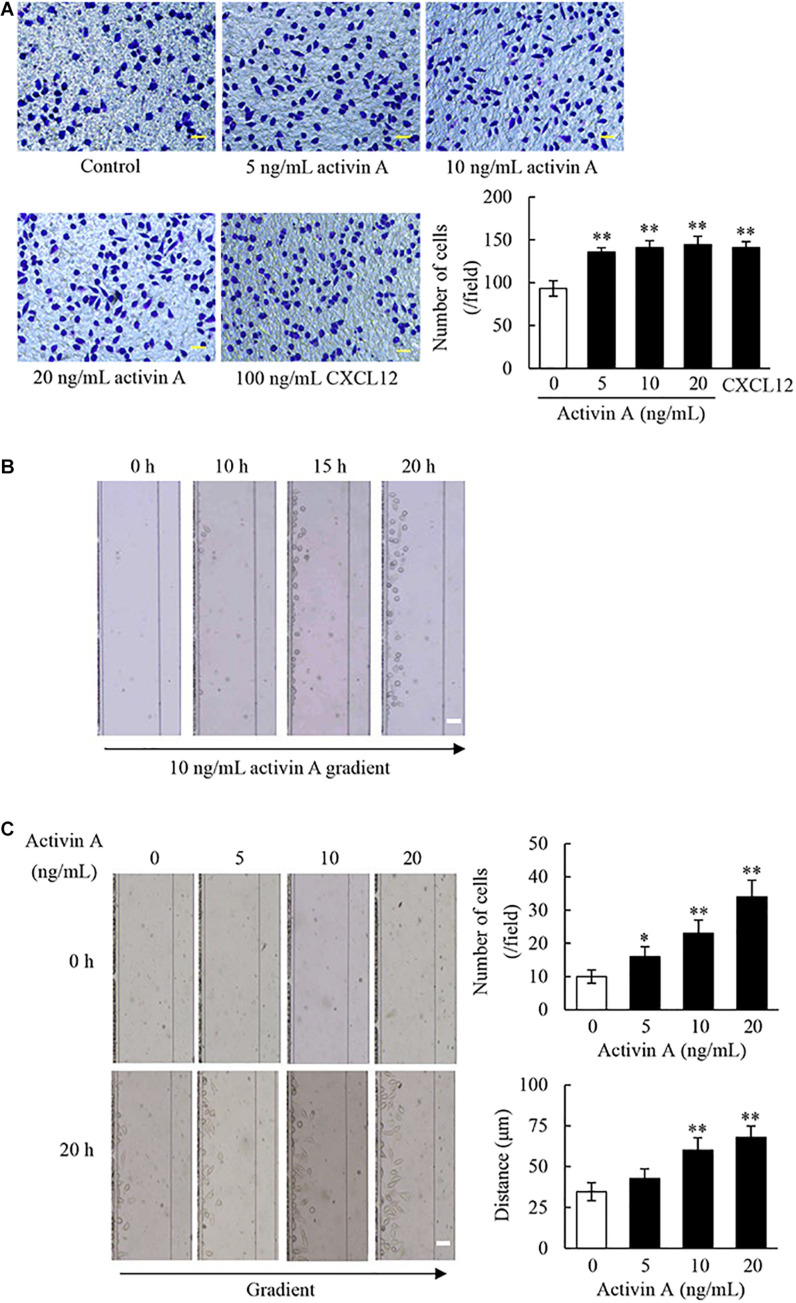
Effect of activin A on migration of L929 cells. **(A)** The chemotactic migration of L929 cells to activin A (0–20 ng/mL) and CXCL12 (100 ng/mL) was analyzed by transwell chamber assay. Cell that passed through porous membrane of the chamber were stained with Giemsa. Scale bar = 50 μm. The graph showed the average number of Giemsa-stained cells in three separate experiments. ^∗∗^*P* < 0.01 compared with 0 ng/mL control group. **(B)** Images of L929 cells migration toward 10 ng/mL activin A gradient at different times were taken in the microfluidic device. Scale bar = 50 μm. **(C)** Images of L929 cells migration toward different concentrations activin A gradient (0–20 ng/mL) were taken in the microfluidic device at 0 and 20 h, respectively. Scale bar = 50 μm. The graph showed the average number and distance of migrating cells in the same size fields of the microfluidic device in three separate experiments. ^∗^*P* < 0.05 and ^∗∗^*P* < 0.01 compared with 0 ng/mL control group.

The migration of fibroblasts to inflammatory sites is critical for wound healing and tissue fibrosis. In the present study, the transwell chamber assay was first carried out to examine effects of activin A on cell migration. The results showed that activin A significantly induced migration of L929 cells in a dose-dependent manner (Figure 2A), in which CXCL12 was used as positive control.

In order to test whether activin A is a chemoattractant to L929 cells, we utilized the D^3^-Chip of microfluidic device to determine the motility and chemotactic migration of L929 cells to the activin A gradients. The results revealed that activin A gradients induced the chemotactic migration of L929 cells in both time-dependent and dose-dependent manner (Figures 2B,C). Activin A gradients increased not only the number of migrating cells, but also the distance of cell migration by the end-point migration distance analysis (Figure 2C). Taken together, the above data indicated that activin A was a chemokine to L929 cells, and suggested that activin A might facilitate tissue repair and fibrosis by inducing the chemotactic migration of fibroblasts to damaged or inflammatory foci.

### Effects of Activin A on Wound Healing in L929 Cells

**FIGURE 3:**
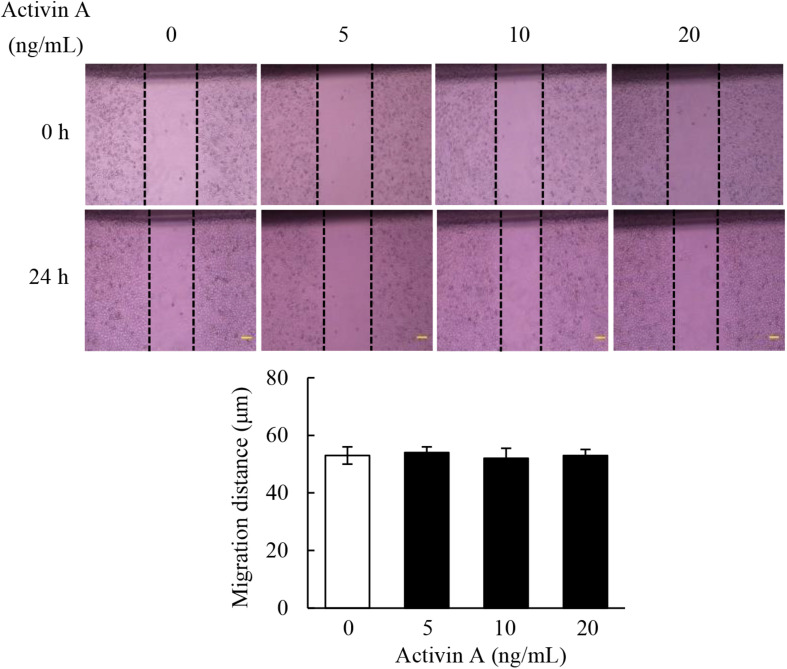
Effects of activin A on wound healing in L929 cells. A scratch-wound was generated in monolayer L929 cells, and then cells were treated with activin A (0–20 ng/mL) for 24 h. The graph showed the degree of wound healing from three separate experiments. Scale bar = 100 μm.

Fibroblasts are the main components involved in wound healing. To examine effects of activin A on wound healing abilities of L929 cell, we performed a scratch wound healing assay in the presence or absence of activin A. As shown in Figure 3, activin A exerted no significant effects on the wound healing of L929 cells *in vitro*, suggesting that although activin A induced the chemotactic migration of L929 fibroblasts, it might not directly affect the horizontal movement of fibroblasts in the wound healing assay that lacks activin A gradients.

### Effects of Activin A on Pro-fibrosis in L929 Cells

**FIGURE 4:**
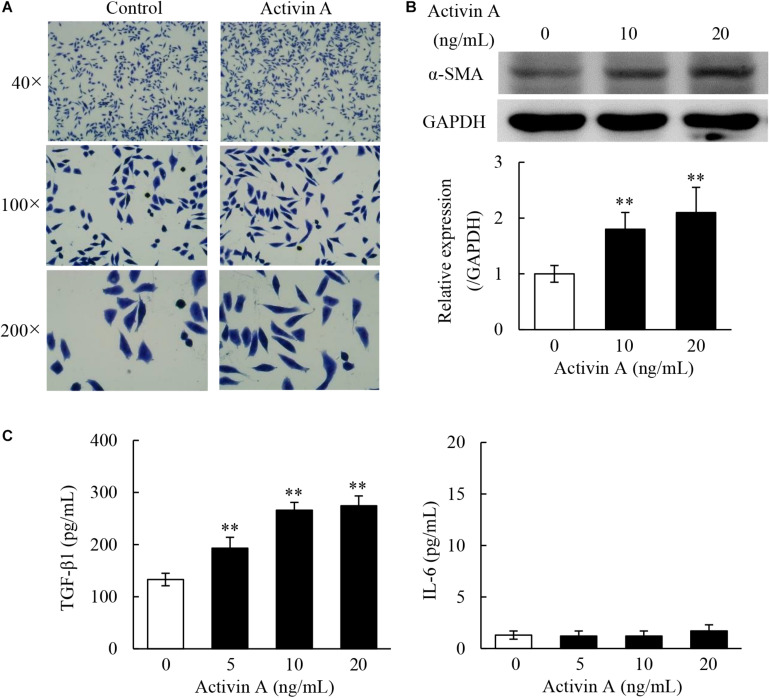
Pro-fibrosis role of activin A in L929 cells. **(A)** Representative images of L929 cells by Giemsa staining. L929 fibroblasts were treated with or without 10 ng/mL activin A for 24 h. **(B)** Western blotting analysis showed that activin A promoted α-SMA expression. The graph represented the relative levels of proteins in three separate experiments. The levels of α-SMA protein were normalized against GAPDH, and the results were shown as the fold-increase of the control. ^∗∗^*P* < 0.01 compared with 0 ng/mL control group. **(C)** L929 cells were incubated containing 0, 5, 10, or 20 ng/mL activin A for 24 h. Levels of TGF-β1 and IL-6 in the supernatant of cultured L929 cells were measured by ELISA. Data represent mean ± SD (*n* = 6). ^∗∗^*P* < 0.01 compared with 0 ng/mL control group.

In the case of tissue repair, fibroblasts can transform to myofibroblasts, that are involved in both fibrogenesis and extracellular matrix (ECM) remodeling stages. To test effects of activin A on pro-fibrosis, we performed Giemsa staining to observe the morphological changes in L929 cells treated with activin A. As shown in Figure 4A, the control L929 cells were spindle or polygonal in shape with short cytoplasmic processes. In contrast, L929 cells treated with activin A mainly adopted a spindle shape with longer cytoplasmic processes. Next, α-SMA, a myofibroblast marker, was assessed by Western blotting. The results showed that activin A treatment of L929 cells increased the level of α-SMA in a dose-dependent manner (Figure 4B). Finally, we used ELISA to measure levels of TGF-β1, a factor closely related to tissue fibrosis, and IL-6, a pro-inflammatory cytokine. As a result, TGF-β1 levels significantly increased in the supernatant of L929 cells treated with activin A, but IL-6 levels did not change (Figure 4C). These results indicated that activin A might not directly trigger the fibroblast-mediated acute inflammation response, but promote fibroblasts-mediated tissue fibrosis.

### Effects of Activin A on Calcium Flux in L929 Cells

**FIGURE 5:**
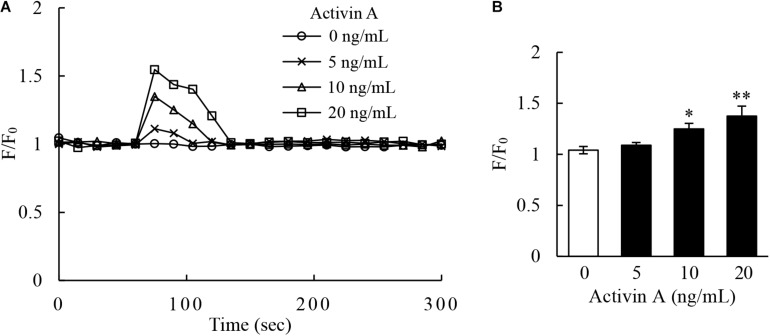
Effect of activin A on calcium signaling of L929 cells. **(A)** Kinetics of calcium signal was measured by the Fluo-4 intensity in L929 cells treated with 0–20 ng/mL activin A. **(B)** The graph showed the peak values of calcium signal upon stimulation under different concentrations activin A. The calcium level was represented by the Fluo-4 signal intensity normalized to the baseline (F/F_0_). The graph represented the results from three separate experiments. ^∗^*P* < 0.05 and ^∗∗^*P* < 0.01 compared with 0 ng/mL control group.

Calcium signaling has long been well-recognized to be associated with the activation of cells including proliferation, apoptosis and migration (Clapham, 2007; Berridge, 2016). In this regard, the Fluo-4-based calcium assay were performed by flow cytometry to examine the levels of intracellular calcium in L929 cells. The results revealed that activin A increased the calcium levels in L929 cells (Figure 5), suggesting that the effects of activin A on L929 cells migration might be associated with calcium signaling.

### Effects of Activin A on ERK Signaling in L929 Cells

**FIGURE 6:**
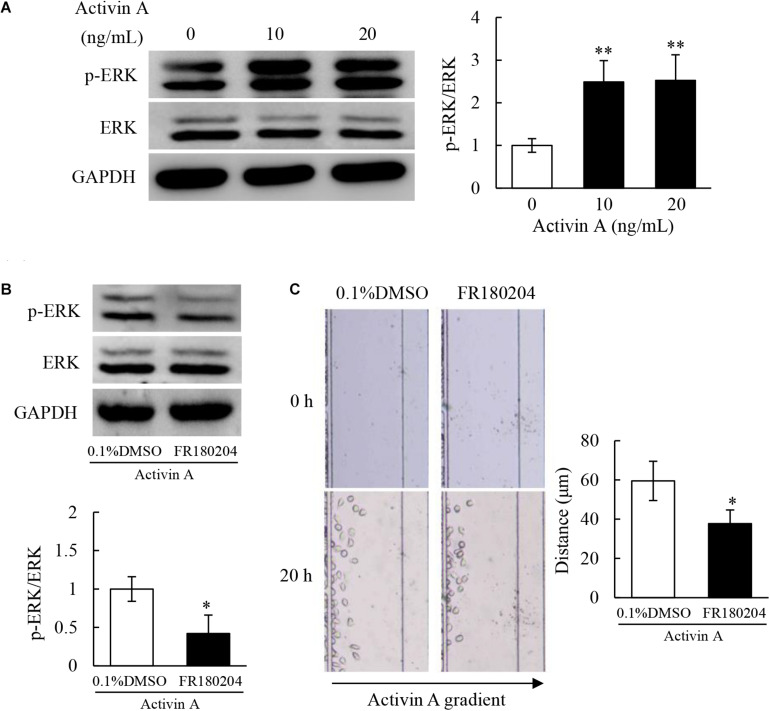
Effects of activin A on ERK signaling in L929 cells. **(A)** Levels of p-ERK and ERK proteins were examined by Western blotting in L929 cells subject to activin A for 2 h. The graph represented the relative levels of proteins in three separate experiments. ^∗∗^*P* < 0.01 compared with 0 ng/mL control group. **(B)** L929 cells were seed in 12-well plates and pretreated for 1 h with 0.1% DMSO or 1 μmol/L FR180204 before treated for 2 h with or without 10 ng/mL activin A. Levels of p-ERK and ERK were examined by Western blotting. The graph represented the relative levels of proteins in three separate experiments. ^∗^*P* < 0.05 compared with 0.1% DMSO control group. **(C)** L929 cells were pretreated for 12 h with 0.1% DMSO or 1 μmol/L FR180204 and then cell migration toward 10 ng/mL activin A gradient was examined by microfluidic devices. ^∗^*P* < 0.05 compared with 0.1% DMSO control group.

Previous study has demonstrated that activin A does not antagonize TNF-α-induced activation of L929 cells via classical Smad signaling pathway (Jiang et al., 2020). Therefore, in order to determine the signal transduction mechanism of activin A regulating the migration of L929 cells, levels of ERK and p-ERK proteins were examined by Western blotting. The results showed that the p-ERK protein levels increased in L929 cells treated with activin A (Figure 6A). Additionally, pretreatment with FR180204 attenuated activin A-induced increase of p-ERK levels (Figure 6B), and decreased activin A-induced migration of L929 cells (Figure 6C). Taken together, the above results indicated that activin A might induce the migration of L929 fibroblasts through ERK signaling.

## Discussion

Fibroblasts represent a heterogeneous cell subset differentiated from embryonic mesenchymal cells, which are spindle or polygonal in shape with round or oval nucleus, and lack epithelial and leukocyte lineage markers (Martin and Blaxall, 2012). Traditionally, fibroblasts are suggested to exert a structural role by synthesizing and remodeling ECM in tissues. Additionally, apart from their role in structural support, fibroblasts also secrete and respond to cytokines to be involved in inflammatory and immune response. Thus, fibroblasts maintain the homeostasis of adjacent cells, orchestrate inflammation, and play an important role in tissue development, remodeling and repair. However, in the case of persistent inflammation, fibroblasts will abnormally proliferate and become activated, resulting in excessive production and deposition of ECM to cause tissue fibrosis (Wynn and Ramalingam, 2012). Therefore, the regulation of fibroblast activities is crucial to the inflammatory process and outcome.

Activin, which is originally extracted from porcine follicular fluid, is a double-chain glycoprotein that stimulates the secretion of follicle-stimulating hormone from pituitary extracts and exerts an important effect on reproductive development (Ling et al., 1986; Vale et al., 1986). So far, at least three bioactive forms of activins are identified, including activin A (βA/βA), activin B (βB/βB) and activin AB (βA/βB), among which activin A is the most widely distributed form with the strongest activity (de Kretser et al., 2002). Besides, the amino acid sequence of activin A in mice shared 100% identity to that in humans (Schwall et al., 1988). Activin A is expressed in a variety of tissues and cells, and exerts a critical regulatory function in inflammation, immunity, tumor, tissue repair and fibrosis (Morianos et al., 2019; Roudebush et al., 2019; Zhang et al., 2019). However, it remains unclear whether activin A affects adhesion and migration of fibroblasts.

Wound repair and tissue fibrosis are related to a series of coordinated processes, such as wound contraction, angiogenesis, cell proliferation and migration, ECM deposition and remodeling. In these processes, various cells are involved, mainly including keratinocytes, fibroblasts and endothelial cells (Gurtner et al., 2008). In the late stage of inflammation or persistent chronic inflammation, the proliferation and adhesion of fibroblasts are the key factors for wound healing and tissue fibrosis (Bainbridge, 2013). Therefore, this study examined effects of activin A on the viability, proliferation and adhesion of fibroblast cell line L929. The results showed that activin A significantly enhanced the adhesion ability of L929 cells, but had no effects on cell viability and proliferation. These findings suggested that activin A profited retention of fibroblasts in the local inflammation tissues.

In the process of tissue repair and fibrosis, fibroblasts can directionally migrate toward the wound sites or inflammation foci. Transwell assay and scratch wound healing assay are the traditional methods to examine cell migration and tissue healing (Tan et al., 2017). Herein, results of scratch wound healing assay showed that activin A had no significant effect on the wound healing of L929 cells *in vitro*, suggesting that activin A might not directly affect the horizontal movement of fibroblasts. However, we found that activin A induced the chemotactic migration of fibroblasts in transwell chamber assay and microfluidic device. The transwell assay evaluates chemotaxis of cells by detecting the number of migrated cells, but it does not determine the migration speed, distance and the specific chemotaxis impact of cytokines on single cells, whereas microfluidic technology is able to detect migration distance and trajectory of single cells (Jia et al., 2019). Indeed, our microfluidic analysis confirmed the chemotactic effect of activin A on L929 cells, including the increased number and distance of migrating cells. Collectively, these data indicated that activin A was a novel chemokine that could induce the directed migration of fibroblasts.

Fibrocytes can be transformed into fibroblasts in the inflammation state. Fibroblasts are spindle or polygonal in shape with abundant cytoplasm and cytoplasmic processes, and they can synthesize and secrete various ECM (Chong et al., 2019). This study found that the number of spindle cells was increased, and the cytoplasmic processes were elongated in L929 cells treated with activin A. These results suggest that activin A may induce the differentiation of fibroblast into myofibroblast, which is the key cellular mediator of fibrosis and can serve as the primary collagen-producing cell when activated (Wynn, 2008). α-SMA is a myofibroblast marker, and over-expressed in the context of organ fibrosis (Duan et al., 2017; Lv et al., 2019). We found that activin A up-regulated the expression of α-SMA in L929 cells, further supporting that activin A might promote the differentiation of fibroblast into myofibroblast. TGF-β1 is not only an important growth and differentiation factor, but also involved in immune response regulation (Delaney et al., 2017). In addition, TGF-β1 plays a key role in the formation of tissue fibrosis in the late stage of inflammation, which is achieved by regulating the phenotype and function of fibroblasts, inducing the differentiation of fibroblasts into myofibroblasts and promoting ECM deposition (Hinz et al., 2012; Kim et al., 2018). This study revealed that activin A promoted the secretion of TGF-β1, but not IL-6 in L929 cells. Typically, IL-6 is an important proinflammatory factor that is mainly produced by the activated T cells, macrophages and fibroblasts. Our findings suggested that activin A might not be involved in the fibroblast-mediated acute inflammatory response, but play a key role in fibroblast-mediated fibrosis.

Activin A, like other members of TGF-β superfamily, can activate Smad signaling pathway to regulate cell activities (Qi et al., 2017). However, previous studies found no significant changes of p-Smad3 level in L929 cells stimulated with activin A (Jiang et al., 2020). As an important second messenger in cells, calcium ion extensively exists in the living organisms and participates in a variety of signaling pathways (Varghese et al., 2019). Calcium signaling is involved in the regulation of cell biological activities, such as cell proliferation, differentiation, apoptosis and migration (Pinto et al., 2015; Tiwari et al., 2017). We previously reported that activin A participated in the regulation of neutrophil migration through calcium signaling (Xie et al., 2017). Our present study revealed that activin A significantly increased the calcium flux in L929 cells, indicating that the biological effects of activin A on L929 cells might be related to calcium signaling. MAPK/ERK signaling pathway is also closely related to wound healing and tissue fibrosis (Kim et al., 2019). Aberrant activation of ERK signaling has been confirmed in systemic organ fibrosis, with ERK phosphorylation modulating fibroblast differentiation (Leivonen et al., 2005; Liu et al., 2020). Besides, MAPK/ERK signaling pathway is also involved in the migration of RA-FLSs (Liu et al., 2018). In this study, we found that activin A induced ERK phosphorylation, whereas ERK inhibitor FR180204 corrected the p-ERK excess and inhibited the activin A-induced migration of L929 cells. Thus, these data indicated that activin A regulated fibroblast activities, such as cell migration, through calcium signaling and ERK signaling.

## Conclusion

In summary, we found that activin A promoted adhesion, induced chemotactic migration of L929 cells by calcium signaling and ERK signaling, and played a pro-fibrosis role by elevating TGF-β1 release. Thus, this study shed new light on understanding the role of activin A in tissue fibrosis.

## Data Availability Statement

The original contributions presented in the study are included in the article/Supplementary material, further inquiries can be directed to the corresponding author/s.

## Author Contributions

ZL and XC designed the experiments. LJ, YQ, and XK performed the experiments. LJ and RW analyzed the data. LJ and ZL drafted the manuscript. JQ, FL, and XC revised the manuscript. All authors read and approved the final manuscript.

## Conflict of Interest

The authors declare that the research was conducted in the absence of any commercial or financial relationships that could be construed as a potential conflict of interest.
